# Evolution of Ozone Pollution in China: What Track Will It Follow?

**DOI:** 10.1021/acs.est.2c08205

**Published:** 2022-12-28

**Authors:** Jia Guo, Xiaoshan Zhang, Yi Gao, Zhangwei Wang, Meigen Zhang, Wenbo Xue, Hartmut Herrmann, Guy Pierre Brasseur, Tao Wang, Zhe Wang

**Affiliations:** † Key Laboratory of Urban and Regional Ecology, Research Center for Eco-Environmental Sciences, Chinese Academy of Sciences, Beijing 100085, China; ‡ University of Chinese Academy of Sciences, Beijing 100049, China; § Division of Environment and Sustainability, The Hong Kong University of Science and Technology, Kowloon 999077, Hong Kong, China; ∥ State Key Laboratory of Atmospheric Boundary Layer Physics and Atmospheric Chemistry (LAPC), Institute of Atmospheric Physics, Chinese Academy of Sciences, Beijing 100029, China; ⊥ Center of Air Quality Simulation and System Analysis, Chinese Academy of Environmental Planning, Beijing 100012, China; # Atmospheric Chemistry Department (ACD), Leibniz Institute for Tropospheric Research (TROPOS), Permoserstraße 15, Leipzig 04318, Germany; ∇ Department of Civil and Environmental Engineering, The Hong Kong Polytechnic University, Kowloon 999077, Hong Kong SAR, China; ○ Environmental Modeling Group, Max Planck Institute for Meteorology, Hamburg 20146, Germany; ◆ Atmospheric Chemistry Observations and Modeling Laboratory, National Center for Atmospheric Research, Boulder, Colorado 80307, United States

**Keywords:** ozone pollution, diagnosis approach, ozone formation regime, ozone−precursor relationship, air pollution mitigation

## Abstract

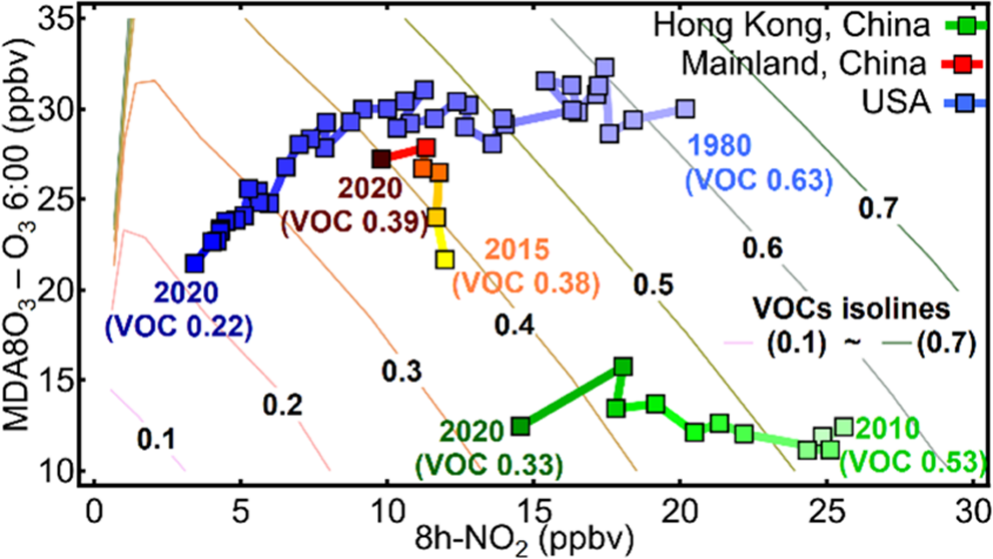

Increasing surface ozone (O_3_) concentrations has emerged as a key air pollution problem in many urban regions worldwide in the last decade. A longstanding major issue in tackling ozone pollution is the identification of the O_3_ formation regime and its sensitivity to precursor emissions. In this work, we propose a new transformed empirical kinetic modeling approach (EKMA) to diagnose the O_3_ formation regime using regulatory O_3_ and NO_2_ observation datasets, which are easily accessible. We demonstrate that mapping of monitored O_3_ and NO_2_ data on the modeled regional O_3_–NO_2_ relationship diagram can illustrate the ozone formation regime and historical evolution of O_3_ precursors of the region. By applying this new approach, we show that for most urban regions of China, the O_3_ formation is currently associated with a volatile organic compound (VOC)-limited regime, which is located within the zone of daytime-produced O_3_ (DPO_3_) to an 8h-NO_2_ concentration ratio below 8.3 ([DPO_3_]/[8h-NO_2_] ≤ 8.3). The ozone production and controlling effects of VOCs and NO*_x_* in different cities of China were compared according to their historical O_3_–NO_2_ evolution routes. The approach developed herein may have broad application potential for evaluating the efficiency of precursor controls and further mitigating O_3_ pollution, in particular, for regions where comprehensive photochemical studies are unavailable.

## Abstract 2

An approach for diagnosing ozone pollution evolution, chemical formation regimes, and responses to precursor controls is developed.

## Introduction

Ozone (O_3_) has been regarded as a principal component of photochemical pollution in urban regions worldwide and has received continuous attention from both the scientific and regulatory communities due to its adverse impacts on human health, air quality, the climate, and the natural environment.^1,2^ Tropospheric O_3_ is produced from the sunlight-initiated photochemical processing of volatile organic compounds (VOCs), nitrogen oxides (NO*_x_*), and, in a condition-dependent manner, carbon monoxide (CO), emitted from a vast variety of sources.^3,4^ Although extensive efforts have been made to regulate O_3_ precursor emissions worldwide,^5,6^ O_3_ concentrations reached very high levels, for example, in North America, before responding to control strategies developed and implemented over several decades.^7,8^ Moreover, O_3_ pollution continues to increase markedly in East Asia.^9−13^ The nonlinear responses of O_3_ formation to precursor emissions represent a major issue regarding O_3_ pollution control, thus posing challenges to the formulation of a universal and efficient O_3_ control strategy in regions with various chemical environments and regimes.^14,15^

Several approaches have been developed and utilized to identify O_3_ formation regimes and their relationships with precursor emissions. These methods include onsite observations of indicator ratios,^16^ emission-based air quality models,^13,17^ relative incremental reactivity (RIR) assessments performed with observation-constrained models,^18^ and the remote sensing of formaldehyde-to-NO*_x_* ratios.^13,19^ Most of these methods require sophisticated measurements, remote sensing data, accurate emission inventories, or detailed speciation information of emitted VOCs, while the O_3_ and NO_2_ data provided by regulatory monitoring networks are typically used to validate modeling results. In this work, we try to explore the utility of easily accessible NO_2_ and O_3_ monitoring datasets and develop an alternative approach analogous to the empirical kinetic modeling approach (EKMA) to obtain a classification scheme for diagnosing O_3_ formation regimes in different regions. By visualizing the site-to-site variations and evolving routes of O_3_–NO_2_ relationships, we shed some light on the efficiency of precursor controls in different regions of China and the development of more cost-effective emission control strategies in the future.

## Materials and Methods

Continuous and standardized NO_2_ and O_3_ monitoring has been carried out at over 1200 sites in China by the National Environmental Monitoring Center (CNEMC) since 2013. This monitoring network provides long-term NO_2_ and O_3_ data at urban and suburban sites, covering different climatic regions of China. Hourly O_3_ and NO_2_ data recorded at national monitoring sites in China from January 2015 to December 2020 were obtained from the monitoring network website (http://106.37.208.233:20035). Historical O_3_ and NO_2_ monitoring data from Hong Kong and the United States were also incorporated into the analysis of this work. O_3_ and NO_2_ data recorded at the Air Quality Monitoring Stations (AQMS) in Hong Kong from 2010 to 2020 were obtained from the Hong Kong Environmental Protection Department (HKEPD) website. O_3_ and NO_2_ data collected by the United States Air Quality System (AQS) network from 1980 to 2020 were obtained from the Environmental Protection Agency (EPA) website. More detailed information on the sources and selection criteria of O_3_ and NO_2_ monitored data are described in the Supporting Information. In this work, we examined the relationship of 8h-NO_2_ with the daytime-produced O_3_ value (DPO_3_ = MDA8O_3_-O_3_ (6:00_LT_)). MDA8O_3_ refers to the maximum daily 8 h average ozone. 8h-NO_2_ is the average NO_2_ in the same 8 h period of MDA8O_3_. Daytime-produced O_3_ value is defined as the difference between the MDA8O_3_ value and the pre-sunrise O_3_ measured at 6:00 am local time in the day. The metric DPO_3_ (MDA8O_3_-O_3_) (6:00_LT_) is used instead of MDA8O_3_ for better conforming to the definition of the O_3_ formation. As shown in Figure S1, the O_3_ diurnal variations indicate small O_3_ production at clean regions, such as the two background sites in Wyoming, US, whereas the MDA8O_3_ levels cannot reflect the small O_3_ local formation at these sites.

A zero-dimensional (0D) photochemical box model based on the Regional Atmospheric Chemistry Modeling (RACM) mechanism^20^ was utilized to simulate the photochemical relationship between DPO_3_ and 8h-NO_2_ in a similar manner to the EKMA application in different regions and cities. We defined a default setting as a typical condition representing the average of meteorological and environmental situations. The default case was run under a moderate-condition setting in China, with assumptions of national average latitude 34 °N, temperature 290 K, relative humidity (RH) 50%, mixing layer height (MLH) varying from 200 to 1000 m, and on date of September 23rd (average solar radiation of a year). The speciation of anthropogenic VOCs (AVOCs) was derived from the Multiresolution Emission Inventory for China (MEIC) 2017 inventory,^21^ which provides the emissions of the top-30 AVOC species with the highest ozone formation potential (OFP). The biogenic VOC (BVOC) emissions were classified into categories of *d*-limonene and other monoterpenes with two double bonds (LIM), monoterpenes with one double bond (API), and isoprene (ISO) categories, according to a previously published speciation scheme.^22^ More detailed information on the model configuration and scenario settings are described in the Supporting Information.

## Results and Discussion

### DPO_3_ and 8h-NO_2_ Trends in China

**Figure 1:**
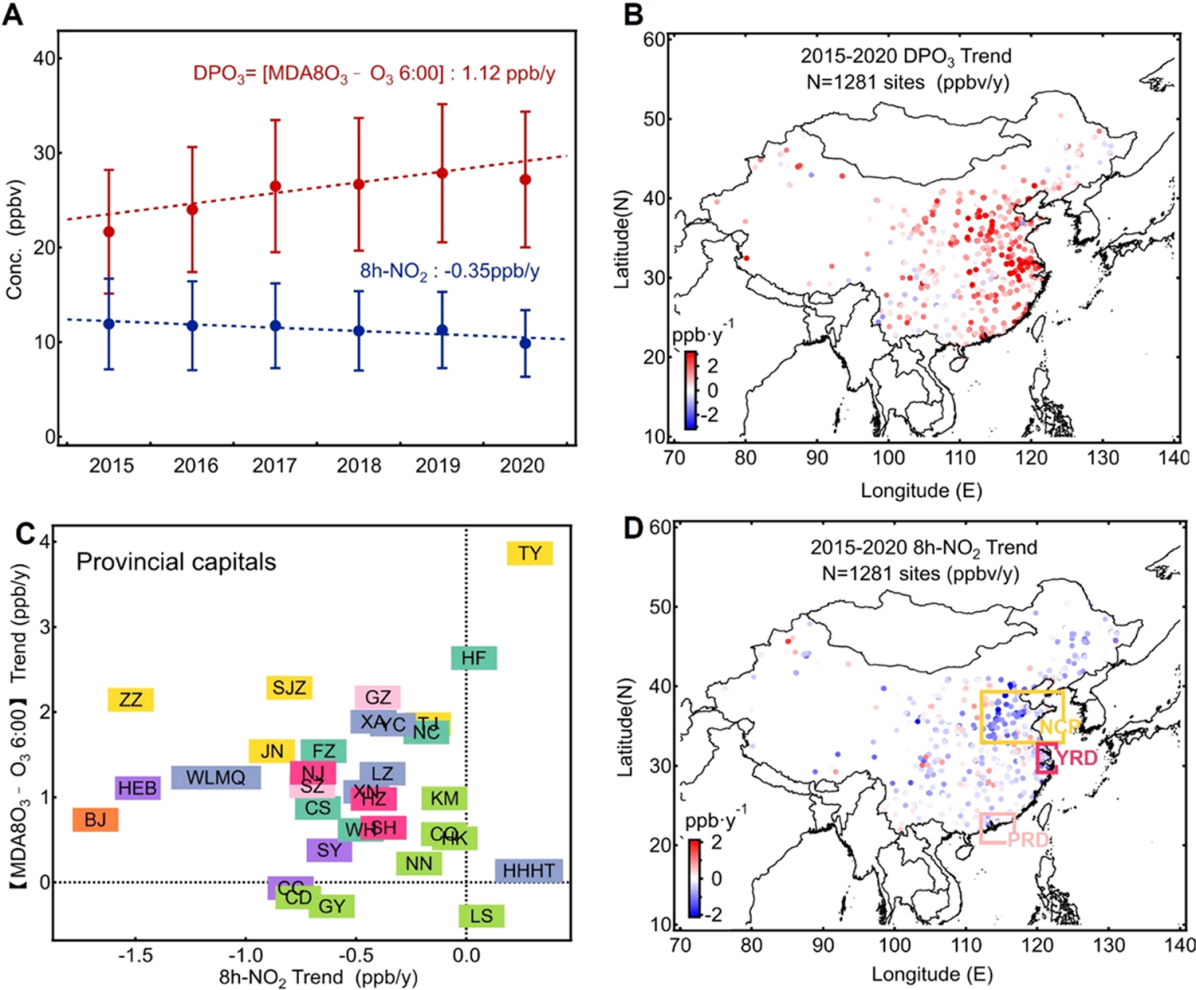
DPO_3_ and 8h-NO_2_ trends in China from 2015 to 2020. (A) Trends and linear regression of the nationally averaged DPO_3_ and 8h-NO_2_ concentrations. Spatial distribution of the annual increase rates of (B) DPO_3_ and (D) 8h-NO_2_ at the national monitoring sites (*N* = 1281) in China for the 2015–2020 period. (C) Quadrant distributions of the DPO_3_ and 8h-NO_2_ change rates in different provincial capital cities in China. The cities were marked using acronyms. The full names of the provincial capital cities and their locations on a map are also provided in Figure S2 in the Supporting Information.

Based on the monitoring dataset of China, we examined the changes in the annual DPO_3_ and corresponding 8h-NO_2_ at a total of 1281 selected CNEMC monitoring sites from 2015 to 2020 (Figure 1). The linearly regressed, nationally averaged increasing rate of DPO_3_ was 1.12 ppb·y^–1^, and the decreasing rate of 8h-NO_2_ was −0.35 ppb·y^–1^ during 2015–2020 (Figure 1A). These results are consistent with previous observations obtained from individual photochemistry projects and those recorded at long-term background monitoring stations, where elevated ground O_3_ levels over China have been widely reported.^9,10,23^

As shown in Figure 1, different cities have reflected different change directions and degrees in their DPO_3_ and 8h-NO_2_ levels. The increase in DPO_3_ and decrease in 8h-NO_2_ were both more noticeable in the cities in the North China Plain (NCP) and Eastern China than elsewhere in the country (Figure 1B,D). Some cities (e.g., Tianjin (TJ)) have experienced a slight 8h-NO_2_ decrease but a large DPO_3_ increase over the past six years, whereas cities such as Chengdu (CD) and Guiyang (GY) have shown minor decreases in DPO_3_ relative to their substantial 8h-NO_2_ reductions (Figure 1C). The O_3_–NO_2_ diagram approach, which was subsequently described, was utilized to further interpret the different O_3_–NO_2_ relationships of these Chinese cities.

### O_3_–NO_2_ Diagram Approach

The “O_3_–NO_2_ diagram approach”, like a transformed EKMA, traces the relationship between the modeled DPO_3_, 8h-NO_2_, and the precursor conditions on the O_3_–NO_2_ diagram modeled from the gas-phase 0D box model. The default case was performed under the typical condition of China. In addition to the default case, different scenario tests were conducted at various latitudes, temperatures, seasons, RH, MLH, and VOC speciation (see the Supporting Information).

**Figure 2:**
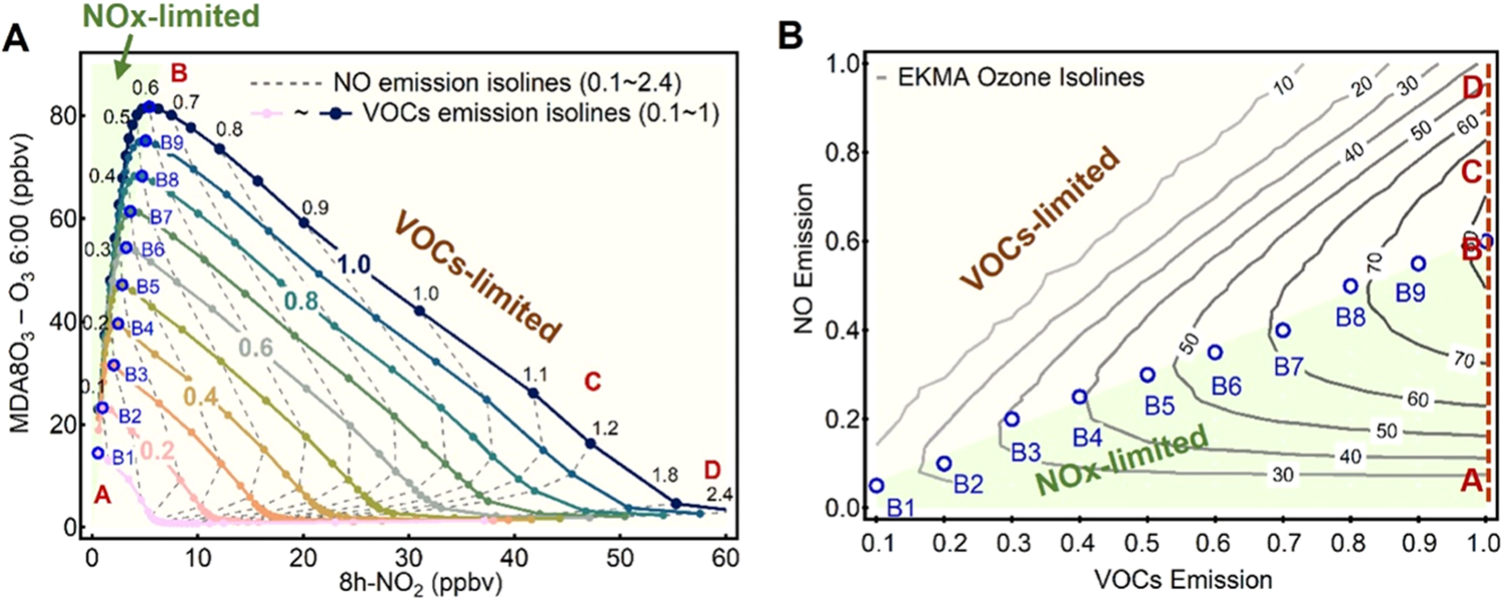
Relationship between DPO_3_ (= MDA8O_3_-O_3_ 6:00) and 8h-NO_2_. (A, left panel) The modeled relationship between DPO_3_ and 8h-NO_2_ follows the A-to-D path along the VOC emission isolines (solid color isolines) with increasing NO*_x_* emissions. Ten VOC emission settings (from 0.3 × 10^–13^ to 3.0 × 10^–13^ mol·cm^–2^·s^–1^ in ten equal intervals) were prescribed and marked with the normalized ratios (0.1–1.0) on the isolines. The corresponding reactivity of VOC emissions, as represented by the equation ∑ Emission-VOC*_i_* × MIR*_i_*, ranged from 0.146 × 10^–10^ to 1.46 × 10^–10^ gram O_3_ cm^–2^·s^–1^. The NO*_x_* emissions (addressed as NO emissions in the model) were prescribed from 0.05 × 10^–12^ to 2.4 × 10^–12^ mol cm^–2^ s^–1^ with 24 different values. Some representative NO*_x_* emission isolines (gray dashed isolines) marked by the normalized ratios (0.1–2.4) are shown in the figure. The modeled data of default case to produce the diagram is listed in Table S3. (B, right panel) The modeled EKMA (empirical kinetic modeling approach) DPO_3_ diagram of the default case, with the corresponding locations of the B points marked on the EKMA diagram. The red dashed line aligns with points A–B–C–D in panel B representing the VOC emission isoline shown in panel A. The O_3_ formation regimes identified as NO*_x_*-limited or VOC-limited are displayed in different background colors in panels A and B.

By representing the calculated DPO_3_ concentrations as the *Y*-values and 8h-NO_2_ as the *X*-values in Figure 2A, the resulting VOC emission isopleth (color lines) depicts how photochemically produced O_3_ and NO_2_ respond to changes in NO*_x_* emissions under fixed VOC emission conditions. The VOC emission isoline reflects the nonlinear response of O_3_ formation to NO*_x_* emissions and reveals a distinct transition of the O_3_ formation sensitivity at turning points (B), at which the DPO_3_ concentration increases (decreases) as NO_2_ increases on the left (right) (Figure 2A). The role of these B points as sensitivity thresholds was also confirmed by their locations on the DPO_3_ isopleth diagram modeled using the traditional EKMA and shown in Figure 2B. NO*_x_*-limited regimes are represented by the left sides of the B points (Figure 2A), where the VOC isolines are densely arranged but do not overlap or cross (Figure S4). These closely spaced paths are consistent with the known insensitivity of O_3_ formation to VOCs under NO*_x_*-limited regimes. In contrast, VOC-limited regimes are represented by the right sides of the B points, with the low-to-high VOC emission isolines arranged from the bottom-left to the top-right (Figure 2A). The slopes of the C-to-B segments of the VOC isolines in Figure 2A represent the extent to which O_3_ increases in response to NO*_x_* mitigation under fixed VOC conditions in a VOC-limited regime.

We also examined the DPO_3_–8h-NO_2_ diagrams modeled under scenarios of different seasons, latitudes, temperatures, relative humidity as well as the VOC speciation (see Figure S5). The VOC isolines tend to be steeper during summer, during high temperatures, and at low latitude, and will be flatter or more inclined if all VOCs are alkenes or aromatics, respectively. In rare cases, the VOC isolines may be bent at the high NO*_x_* emission ends (Figure S5); this condition is dependent on the time course of the NO+NO+O_2_ = 2NO_2_ reaction complementing the suppressed photochemical processes (Figure S6).

**Figure 3:**
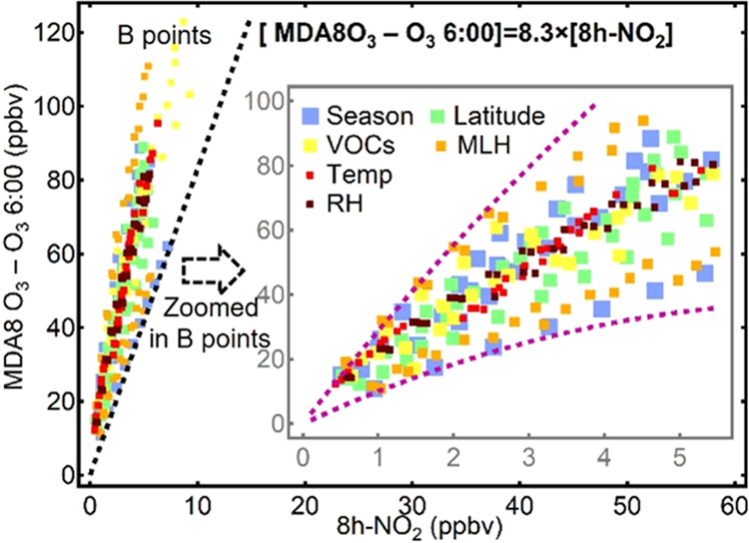
Sensitivity of the locations of the B points to the temperature, RH, season, latitude, MLH, and VOC speciation conditions. The black dashed line, represented by [DPO_3_] = 8.3 × [8h-NO_2_], indicates a safe boundary for VOC-limited regimes under various scenarios in China. The inset shows the expansion of the regime-transition region. The purple dashed lines represent the upper and lower bounds of the regime-transition region with regards to the annually averaged DPO_3_ and 8h-NO_2_ data characterizing Chinese cities, which were modeled under the southmost conditions (20 °N, 303 K, MLH 100–700 m) and northmost conditions (50 °N, 273 K, MLH 400–1000 m), respectively.

The positions of the division points (B) are important for distinguishing among different O_3_ formation regimes. The influence of environmental factors (seasons, latitudes, temperatures, RH, VOC speciation, etc.) on the division point (B) locations was examined. The division points (B) of all of the examined Chinese scenarios were located in a quite narrow area when considering the possible factor ranges characterizing Chinese cities (Figure 3), thus revealing the possibility of identifying the O_3_ formation regime by directly referring to these B point locations. In the figure, the linear equation of [DPO_3_] = 8.3 × [8h-NO_2_] represents the “safe” boundary, indicating a VOC-limited regime with regard to the annual mean O_3_–NO_2_ relationship for most Chinese cities. For more accurate analysis, it is recommended that a localized DPO_3_–8h-NO_2_ diagram should be produced for a specific region or city, with the specialized meteorological condition and VOC speciation of this region/city.

### O_3_ Formation Regimes and Historical Routes Response to Precursor Controls

**Figure 4:**
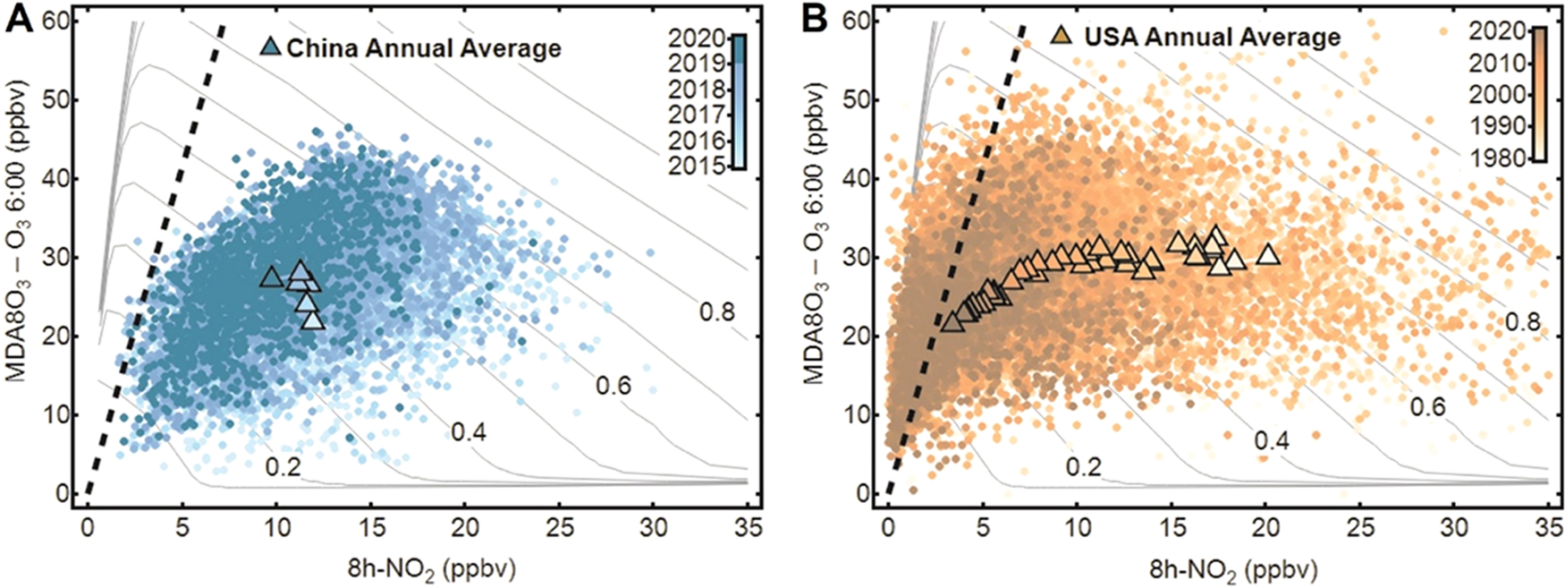
Annual DPO_3_–8h-NO_2_ data and evolving trends in China and USA. Locations of the annually averaged DPO_3_–8h-NO_2_ data recorded at (A) 1281 sites in China from 2015 to 2020 and at (B) monitoring sites in the USA from 1980 to 2020, superposed on the VOC emission isolines (0.1–1.0) derived under the default modeling conditions shown in Figure 2. The dots represent the annual average values at monitoring sites in China or the USA, and the triangles represent the national annual mean values at all sites in different years. The color scales indicate the year in which the data were measured.

**Figure 5:**
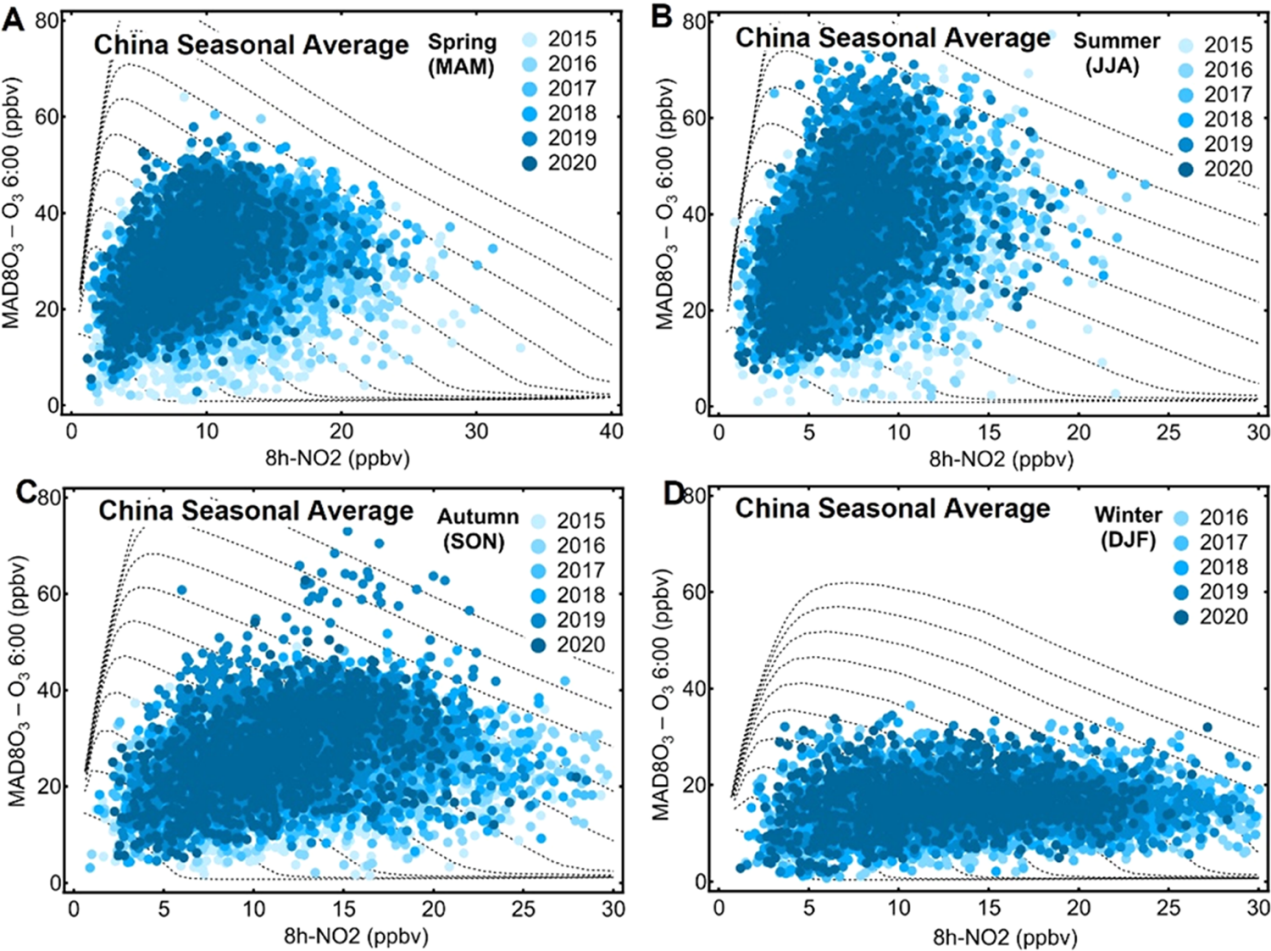
Graphs illustrating the locations of seasonally averaged DPO_3_–8h-NO_2_ data recorded at 1281 sites in China from 2015 to 2020 on modeled seasonal DPO_3_–8h-NO_2_ diagrams.

We mapped the annually averaged DPO_3_ and 8h-NO_2_ data of the Chinese monitoring sites on the modeled O_3_–NO_2_ relationship diagram (Figure 4A). As shown in the figure, most of the measurement data were located to the right of the safe regime-transition boundary line [DPO_3_] = 8.3 × [8h-NO_2_], indicating VOC-limited O_3_ formation regime. We also mapped the seasonal averaged DPO_3_ and 8h-NO_2_ data on modeled seasonal O_3_–NO_2_ relationship diagrams (Figure 5), which show similar results to the yearly averages. The patterns of the monitoring scatters and the predicted seasonal diagrams consistently depicted the varying characteristics of temperature and solar radiation across the seasons. Both the isolines and scatter distribution were steeper in the summer and flatter in the winter.

Previous measurements and modeling studies have generally suggested that ozone production is under VOC-limited regimes in urban and industrial regions but under NO*_x_*-limited regimes in most rural areas in China.^9,13,24,25^ The present work tends to suggest the dominance of VOC-sensitive O_3_ formation regimes in the regions represented by the national monitoring stations. The DPO_3_–8h-NO_2_ scatterplot exhibits an evolving trend toward the upper-left direction with annually increasing O_3_ values during the 2015–2020 period (Figure 4A). This evolution direction is consistent with the NO*_x_* emission control efforts enacted in the country over the past five years. But referring to the location of the modeled safe boundary line [DPO_3_] = 8.3 × [8h-NO_2_], most of the Chinese sites still have a long way to go to reach NO*_x_*-limited regimes though the significant NO*_x_* emission control.

The United States (US) has suffered from high O_3_ pollution for a long time, and the successful emission reductions in recent decades can provide insights into the potential evolution and control of O_3_ pollution.^7,26^ The annual average O_3_ production and NO_2_ data recorded by the US EPA monitoring network from 1980 to 2020 are depicted in Figure 4B, which shows the overall historical route of the USA’s DPO_3_–8h-NO_2_ in the past few decades. The data clearly show an evolution toward the lower DPO_3_ and NO_2_ region moving toward the NO*_x_*-limited regime. More sites have passed the transition point in recent years. Notably, concurrent decreases in O_3_ and NO_2_ were observed when the 8h-NO_2_ value reached approximately 10 ppbv after 2000, but this should not be interpreted as the turning point to the NO*_x_*-limited regime. Instead, these decreases are the result of the simultaneous successful control of NO*_x_* and VOC emissions. By comparing the O_3_ evolution route with the modeling results shown in Figure 4B, the USA data exhibit a shift across approximately five VOC isolines since 1980. An estimated 65% reduction in VOCs and a 70% reduction in NO*_x_* can be inferred based on the locations at which the VOC and NO*_x_* isolines cross. According to the US EPA, national emissions (excluding biogenic and wildfires) of VOCs and NO*_x_* were reduced by 60 and 70%, respectively, from 1980 to 2020 (https://www.epa.gov/air-trends/air-quality-national-summary). Though this semiquantitative estimation is associated with many uncertainties, including VOC speciation and meteorological condition differences between the countries, the trends and changing degrees estimated from the diagram generally match the emission inventory well.

**Figure 6:**
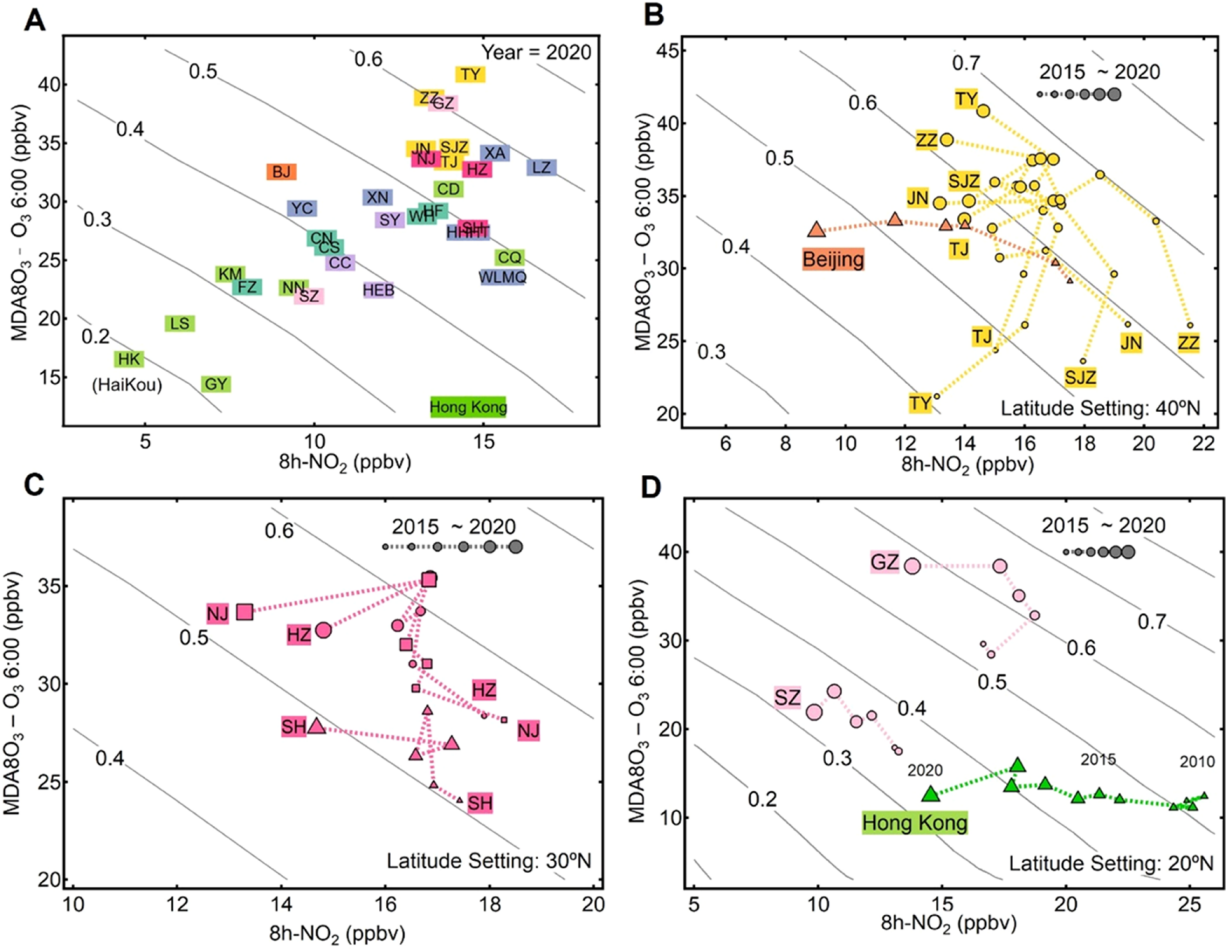
Annual DPO_3_–8h-NO_2_ locations and evolving trends in different regions and cities of China. (A) Annually averaged DPO_3_–8h-NO_2_ data in all provincial capital cities in China in 2020 superposed on the VOC emission isolines established under the default case. The cities are marked using acronyms and grouped in different colors, according to their locations in different regions of China, as in Figures 2 and S2. (B–D) Evolution of DPO_3_–8h-NO_2_ over the past decade in cities located in the major regions of China, superposed on the VOC emission isolines obtained from the average condition of each region. The coverage of the NCP, PRD, and YRD regions is described in Figure S7 in the Supporting Information. The marker size represents the annual data values obtained in different years.

In another direct and useful application of the diagram, we visualized the site-to-site variations and evolution routes of different cities/regions on the DPO_3_–8h-NO_2_ diagram (Figure 6). The locations of the Chinese provincial capitals on the diagram (Figure 6A) are consistent with current knowledge regarding the spatial distributions of NO*_x_* and VOC pollution in China.^27,28^ The highly industrialized and populated cities (e.g., cities in NCP, shown with a yellow background) are located in the upper-right quadrant of Figure 6A, suggesting that this highly polluted region experiences concurrently high NO*_x_* and VOC emissions. In contrast, the relatively less-industrialized cities in southwestern China (green background) are generally located in the bottom-left quadrant of the diagram.

The evolving routes of different cities/regions shown on the diagram shed light on the historical precursor control strategies enacted in these cities/regions. As an example, we compared the evolving routes of the major cities in three most-developed regions in China shown in Figure 6B–D. The NCP, PRD, and YRD cities mostly showed steep DPO_3_–8h-NO_2_ tracks moving along the VOC emission isoline direction. Beijing and Hong Kong presented a relatively flat trace, crossing more VOC isolines and shifting leftward with a large 8h-NO_2_ decrease but a small O_3_ increase. This shift of Beijing toward the lower VOC isolines on the diagram suggests a reduction in VOC emissions of approximately 24% from 2015 to 2020. The O_3_ pollution in Hong Kong is shown to be primarily VOC-limited, exhibiting an O_3_ increasing trend overall throughout the last 20 years.^5^ As shown in Figure 6D, the annual DPO_3_–8h-NO_2_ data recorded in Hong Kong from 2010 to 2020 moved leftward, exhibiting a slight increase in O_3_ production but crossing two VOC emission isolines. This trend implies an approximate VOC reduction of 22% and a NO*_x_* reduction of 24% from 2010 to 2018 in Hong Kong. This estimation agrees well with the emission inventory from HKEPD, in which 26 and 23% reductions during this period were, respectively, reported in VOCs and NO*_x_* emissions,^29^ though the estimation was made based on the assumption of default VOC speciation of China. We also compared the estimated precursor emissions at the 1281 sites from the DPO_3_–8h-NO_2_ diagram with the emission rates obtained from the MEIC for the same region (Figure S8). The general trend of the inferred emission conditions was consistent with the bottom-up emission inventory, thus imparting confidence in the capability of the DPO_3_–8h-NO_2_ approach for diagnosing O_3_ formation and precursor controls in different regions.

It should be noted that this diagram approach would perform better on long-term historical analysis. This is because the utilized long-term observation dataset to some extent can overcome the short-time fluctuations and provide an overall diagnosis of the O_3_–precursor relationship. For example, interannual variations of meteorology would impact the evolving trace of the monitoring data. In addition, the fast reduction in PM_2.5_ concentrations in China in recent years could cause the near-surface radiation increase and reduce the heterogeneous aerosol sink of HO_2_ radicals,^30,31^ thus contributing to O_3_ concentration increases and upward shifting of the DPO_3_–8h-NO_2_ data on the diagram. These factors cannot be distinguished from the modeled DPO_3_–8h-NO_2_ diagram and would bias the diagram-estimated emission changes over the years. In estimating the trend in precursors, long-term datasets have an advantage due to their ability to overcome fluctuations between years.

### Future O_3_ Pollution Mitigation in China

**Figure 7:**
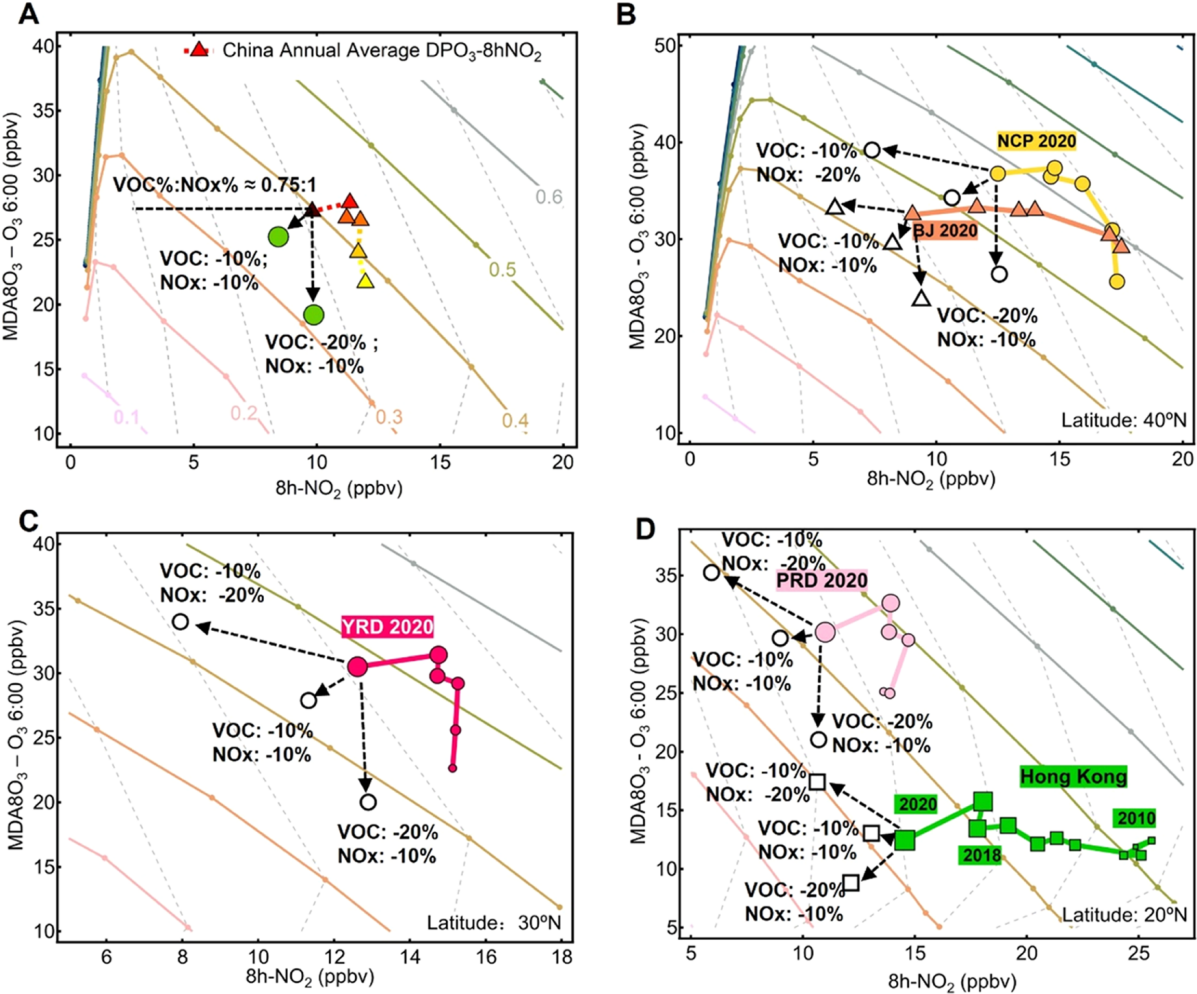
Prediction of DPO_3_–8h-NO_2_ changes under different VOC and NO*_x_* emission control scenarios in China after 2020. (A) Estimated future changes in the national annual average DPO_3_–8h-NO_2_ level in China and (B–D) regions of NCP, YRD, and PRD in China under different emission–reduction scenarios in VOC and NO emissions. The regionally averaged data are superimposed on the VOC emission isolines (solid color lines) and NO emissions isolines (gray dashed lines) derived under the corresponding average condition of each region. The predicted locations and evolving traces of the annual DPO_3_–8h-NO_2_ levels under different VOC/NO*_x_* control strategies or in different regions are shown in open markers and dashed arrows on the diagrams.

In view of the severe O_3_ pollution in China, the government has implemented and planned aggressive control measures regarding the emissions of pollutant species. Thus, we evaluated the impacts of future precursor controls on O_3_ pollution using the diagram estimation approach (Figure 7). The approach shows that a minimum reduction ratio (0.75:1.0) for VOC/NO*_x_* is required to achieve nonincreasing O_3_ production from the current annual levels. China′s 14^th^ five-year plan calls for reductions of more than 10% in both VOC and NO*_x_* emissions by 2025 and emphasizes that the VOC reduction ratio should be no less than that of NO*_x_* in polluted regions. This synergetic 10% reduction in VOC and NO emissions is estimated to decrease the photochemically produced annual O_3_ concentration by approximately 2.0 ppbv (Figure 7A) and the summer O_3_ production by 1.5 ppbv on the national average (Figure S9). Based on the locations of the present DPO_3_–8h-NO_2_ and the localized baseline for the NCP, YRD, and PRD regions, regional reduction effects were further investigated (Figure 7B–D). With a synergetic 10% reduction in both VOC and NO*_x_* emissions, the anticipated decreases in DPO_3_ would be 2.5, 2.6, and 0.5 ppbv for the NCP, YRD, and PRD regions, respectively. On the contrary, DPO_3_ in Hong Kong may increase by 0.6 ppb under the same reduction scenario and will reduce only when a higher VOC reduction percentage can be achieved. The estimated DPO_3_ levels will fall by 10.4, 10.5, and 9.2 ppbv in NCP, YRD, and PRD, if the NO*_x_* and VOC emissions can be reduced by 10 and 20%, respectively. For the cases with only VOCs decreased by 20%, the predicted DPO_3_–8h-NO_2_ points would move downward along the NO*_x_* isolines (gray dash lines in Figure 7), with the DPO_3_ drops of 16.8, 16.3, 14.5, and 6.1 ppbv in NCP, YRD, PRD, and Hong Kong, respectively; meanwhile, the 8h-NO_2_ level would likely rise.

Despite the fact that the O_3_–NO*_x_*–VOC sensitivity could vary across regions and seasons, the majority of recent studies suggest that VOC-targeted management is a more workable solution in China. For example, based on the WRF-CMAQ modeling, Wang et al. proposed that O_3_ pollution mitigation in NCP, YRD, and PRD would be effective when the VOCs/NO*_x_* reduction ratio is more than 2:1.^32^ Another modeling study in PRD showed that a reduction ratio of VOC/NO*_x_* more than 1:1 was necessary to accomplish synergetic control, and the best O_3_ reduction was found for a VOC-only control scenario.^33^ A recent study based on satellite retrievals also suggested that the ozone concentration in Beijing, Chengdu, and Guangzhou would be significantly lowered if the reduction ratio of VOCs/NO*_x_* is between 2:1 and 4:1.^34^ These previous investigations, together with the present work, all highlighted that the basis for O_3_ pollution management is an approximately 1:1 synergetic reduction of VOC and NO*_x_* and that a ratio greater than 2:1 could contribute to significantly reduced O_3_ levels.

In summary, the good performance on estimating the historical precursors controlling in the US and Hong Kong provides supporting evidence for the applicability of the DPO_3_–8h-NO_2_ diagram when addressing O_3_ pollution and evolution in different regions. This robust and rapid classification approach, in which only the continuous NO_2_ and O_3_ measurement data were utilized, may have broad application potential in evaluating the precursor control effects and assisting in developing O_3_ pollution mitigation strategies, in particular, for regions where comprehensive photochemical studies are unavailable. The historical evolution of air pollution in the US indicates that successfully controlling O_3_ pollution is possible. Synergetic VOC and NO*_x_* reduction and increasingly strict anthropogenic VOC control should be the primary focus at the present stage for controlling O_3_ pollution in China.

## Supporting Information Available

Including monitoring data sources, modeling settings, the data for default case isolines plotting (Table S3), other supporting figures (Figures S1–S9), and tables (Tables S1 and S2) (PDF)

## Notes

All data are available in the main text or Supporting Information. Correspondence and requests for further materials should be addressed to X.Z. (zhangxsh@rcees.ac.cn) and Z.W. (z.wang@ust.hk).
