# Supplementary material for: Evolution of Ozone Pollution in China: What Track Will It Follow?
Source: Environ Sci Technol. 2022 Dec 28;57(1):109–17. doi: 10.1021/acs.est.2c08205 (PMC9835882; doi:10.1021/acs.est.2c08205)
Supplement: Supplementary file 1 — es2c08205_si_001.pdf [file es2c08205_si_001.pdf]

Supplementary Information for

The evolution of ozone pollution in China: what track will it follow?

Jia Guo[1, 2, #], Xiaoshan Zhang[1, 2, *], Yi Gao[2, 4], Zhangwei Wang[1, 2], Meigen Zhang[2, 4], Wenbo Xue[5], Hartmut Herrmann[6], Guy Pierre Brasseur[7,8,9], Tao Wang[7], Zhe Wang[3,*]

[1] Key Laboratory of Urban and Regional Ecology, Research Center for Eco-Environmental Sciences, Chinese Academy of Sciences; Beijing 100085, China.

[2] University of Chinese Academy of Sciences; Beijing 100049, China.

[3] Division of Environment and Sustainability, The Hong Kong University of Science and Technology; Hong Kong, China.

[4] State Key Laboratory of Atmospheric Boundary Layer Physics and Atmospheric Chemistry (LAPC), Institute of Atmospheric Physics, Chinese Academy of Sciences; Beijing 100029, China.

[5] Center of Air Quality Simulation and System Analysis, Chinese Academy of Environmental Planning; Beijing 100012, China.

[6] Atmospheric Chemistry Department (ACD), Leibniz Institute for Tropospheric Research (TROPOS); Permoserstraße 15, Leipzig 04318, Germany.

[7] Department of Civil and Environmental Engineering, The Hong Kong Polytechnic University; Hong Kong SAR.

[8] Environmental Modeling Group, Max Planck Institute for Meteorology; Hamburg 20146, Germany.

[9] Atmospheric Chemistry Observations and Modeling Laboratory, National Center for Atmospheric Research; Boulder, CO 80307, USA.

Present Address:

J.G.: The Hong Kong University of Science and Technology, Hong Kong, China

***Correspondence to: Xiaoshan Zhang (zhangxsh@rcees.ac.cn) and Zhe Wang (z.wang@ust.hk)**

This 19 pages SI file includes 9 figures and 3 tables.

Supplementary Information Text

Materials and Methods

1.1 Monitoring data

Hourly O_3 and NO_2 data recorded at national monitoring sites in China from January 2015 to December 2020 were obtained from the China Ministry of Ecology and Environment (MEE) monitoring network website (http://106.37.208.233:20035). Raw concentration data was reported in microgram per cubic meter (μg/m^3), and was converted to ppbv under the reference temperature of 273 K from January 2015 to August 2018 and 298 K from September 2018 to December 2020. Moreover, raw data was given in Beijing Time, which was converted to local time (LT) based on the longitudes of the sites to get the pre-sunrise O_3 at $6{:}00_{LT}$ of sites. O_3 and NO_2 data recorded at the Air Quality Monitoring Stations (AQMS) in Hong Kong from 2010-2020 was obtained from the Hong Kong Environmental Protection Department (HKEPD) website (https://cd.epic.epd.gov.hk/EPICDI/air/station/?lang=en). O_3 and NO_2 data collected by the United States Air Quality System (AQS) network were obtained from the Environmental Protection Agency (EPA) website (https://aqs.epa.gov/aqsweb/airdata/download_files.html), and the data were reported in ppbv with both information of UTC and LT.

MDA8O_3 is the maximum daily 8h average O_3 concentration, and 8hNO_2 is the average NO_2 concentration of the same 8 hours of MDA8O_3 in the day. Daytime-produced-O_3 value is defined as the difference between MDA8O_3 and the pre-sunrise O_3 at $6{:}00_{LT}$. Some criteria were applied to select the available data and sites used for analysis. Specifically, only the 8h average data with at least 6 hours data available in the dataset were considered as valid 8h average data; only those days with at least 14 valid 8h-moving average data in 8:00-24:00 were considered as a valid day;

the sites with annual data completeness higher than 75% were considered as a valid site. A total of 1281 sites in China with all 6-year data that fulfill the selection criteria were adopted for further analysis. The locations of the provincial capitals of China are given in Fig. S1.

1.2 Modeling settings

1.2.1 Model mechanism and configuration

A 0-D photochemical box model based on the Regional Atmospheric Chemistry Modeling (RACM) mechanism was utilized to simulate the gas-phase photochemistry of O_3 formation. The source code of this model was downloaded from the following website: https://capram.tropos.de/, and only gas-phase reactions were employed in our modeling simulation. The RACM model was devised by Stockwell 1997 [1], which is a lumped mechanism with the definition of VOCs categories taken from Middleton et al. [2].

The model could be performed under user-defined parameters of temperature, relative humidity, day of the year, latitude, and mixing layer height (MLH). The initial concentrations of species were set at their typical nocturnal level under urban condition, and their subsequent concentrations of the following five days were calculated by emission, deposition, and gas-phase reactions simulated in the box model. The VOCs and NOx emissions were calculated by dividing their emission rates (mol cm^{-2} s^{-1}) by boundary layer height (cm). The MLH varied between 200 m and 1000 m from nighttime to daytime. No dilution effect of species was associated with the time-varying boundary layer in the model. The MLH was only counted in the emission and deposition rate calculations. The deposition velocities of species were adopted from Ganzeveld et. al [3], while those species without data given in the reference were assumed the same as the deposition velocity of CO.

Photolysis rates in the box model were calculated for clear sky conditions. No surface albedo or absorption of aerosols was considered. No diurnal variations for the temperature, relative humidity and emission per unit area (mol cm^{-2} s^{-1}) were considered in the simulation. A 5-day model run was performed, and the last day results were employed as the modeling output.

1.2.2 VOCs speciation and scenarios setting

The VOC species considered in this work include anthropogenic VOCs (AVOCs) and biogenic VOCs (BVOCs). The speciation of AVOCs was derived from the Multi-resolution Emission Inventory for China (MEIC) 2017 inventory [4], which provides the emissions of the top-30 most popular AVOC species. MEIC suggested that the CO and the NMVOC emissions in China in 2015 were 153.6 tg/y, and 30.3 tg/y, respectively [5]. Considering the molecule mass of CO is smaller than most NMVOC species, the emission rate of CO in this work, in the unit of mol/cm^2/s, was set as 10 times the total AVOCs emission rate. Wang et al. [6] evaluated the total BVOCs emission was about 35.5 tg/y in 2016 in China. Considering the CNEMC monitoring sites mostly located in urban regions, the BVOCs emission rate in this work was set to be 0.5 times of the AVOCs. The BVOCs emission was classified into categories of *d*-limonene and other monoterpenes with two double bonds (LIM), monoterpenes with one double bond (API), and isoprene (ISO) categories referring to Guenther et al. [7] . The VOCs speciation in the model set is given in detail in Table S1. To quantify the VOCs by their total potentials on O_3 production, we used their corresponding reactivity given by Σ Emission-VOCi ×MIRi (Maximum Incremental Reactivity) in the emission setting. The fraction of the corresponding reactivity (Emi-VOCi×MIRi) of different VOC groups was shown in a pie chart plot as Fig. S2.

The default case was run under a moderate condition setting of China. It assumes the site locating at the latitude of 34°N, which is about the middle latitude of cities in China. The modeling date was set on September 23rd, which has a moderate solar radiation condition of a year. Other settings include: temperature of 290K, relative humidity of 50%, and MLH varied between 200 m and 1000 m in a day. The scenario tests examined the impacts on modeling results of latitude (20°N, 30°N, 40°N, 50°N), temperature (273K, 283K, 293K, 303K), day of the year (March 23rd, June 23rd, September 23rd, December 23rd rd), relative humidity (30%, 20%, 70%, 90%), MLH (100m~700m, 100m~1000m, 400m~700m, 400m~1000m), and the VOCs composition (only alkanes, only alkenes, only aromatics, and only biogenics). The scenario tests were conducted with other factors maintained as the default case and only the examined factor adjusted in each testing run. In the default case and each scenario simulation, the reactivity of VOC emissions represented by the ΣEmi-VOCi×MIRi, varied from 1.46×10^{-11} to 1.46×10^{-10} gram O_3 $cm^{-2}\cdot s^{-1}$ in ten equal intervals, and the NO emission ranged from 5×10^{-14} to 2.4×10^{-12} mol cm^{-2} s^{-1} with 24 different values. These model setting resulted in 240 runs of "NO × VOCs" emission combinations in each case, allowing the evaluation of the O_3 formation regime over a wide range of precursors conditions.

1.3 Uncertainties

The uncertainty of the model result comes from the propagation of uncertainties in different parameters and processes, and the bias of condition settings from the real condition. Previous studies have suggested that the uncertainties in the RACM mechanism in predicting urban O_3 production varied between 20~40%, including the uncertainties from the measurements used to constrain the model, the kinetic rate coefficients, and the product yields of chemical reactions in the RACM mechanism [8]. Besides, considering the differences in geographical location and environmental factors of various cities, uncertainties would be introduced by comparing the DPO_3-

8hNO_2 measurement data in different cities on the same default case diagram. For each region/city, a localized DPO_3-8hNO_2 diagram should be produced in different seasons for more detailed and accurate analysis. For the latitude and temperature ranges of Chinese cities, the most extreme differences between the estimated precursor levels from the default case diagram isolines and the respective isolines of each city, were determined to be lower than 83% for VOCs and 48% for NO, within the 8h-NO_2 range of 5~30 ppbv and DPO_3 of 10~50ppbv. Moreover, the different VOC speciation and temporal changes will also lead to non-negligible uncertainties. Additional uncertainties also come from the ignorance of the meteorological or transport processes in the box model, which was not estimated here.

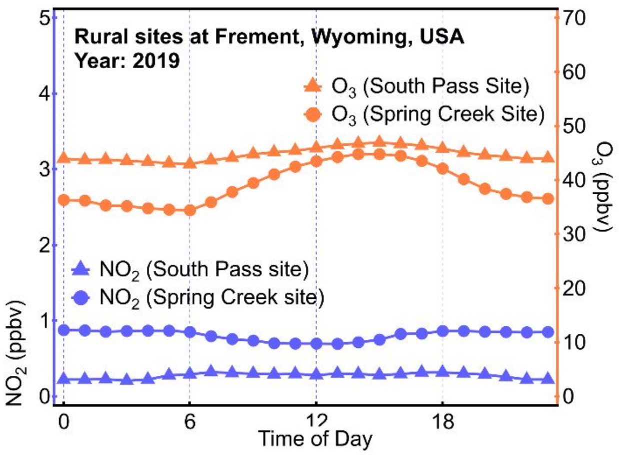

Fig. S1. Daily profiles of NO_2 and O_3 at two rural sites in Wyoming USA, 2019.

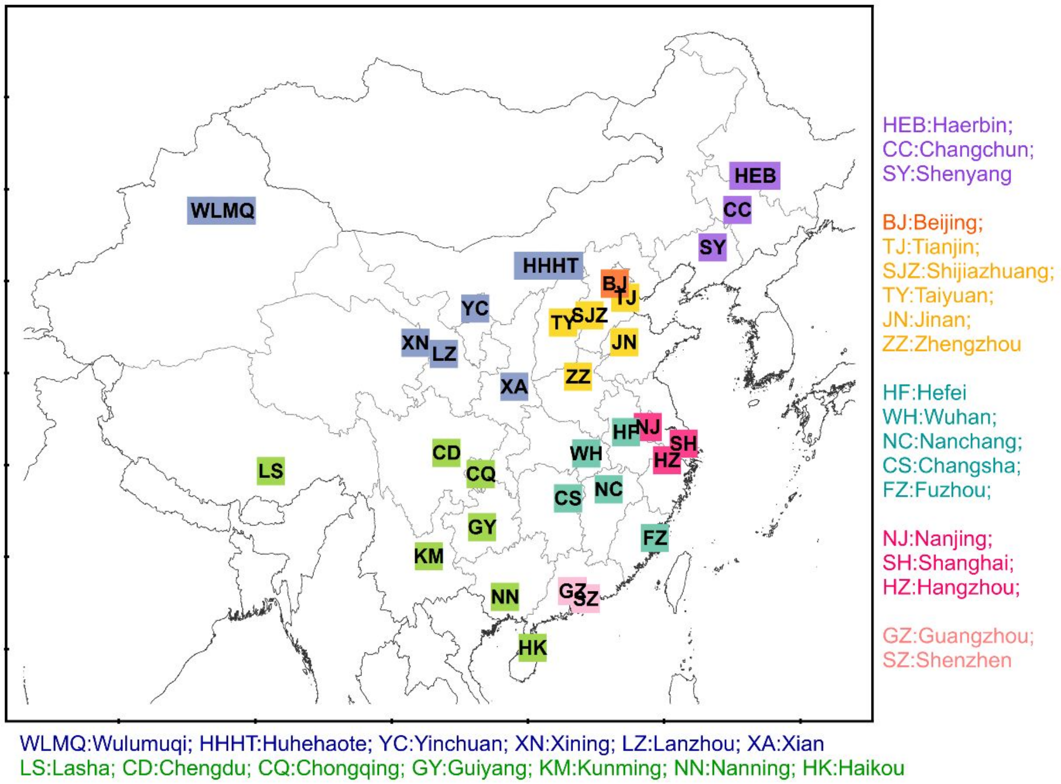

Fig. S2. The locations of the provincial capital cities of China. Cities were marked by the acronym of their names on the map, and full names are shown on the right and bottom of the figure. The colors of the cities are grouped according to their locations in different regions of China, including North East (purple color), Beijing (orange color), North China Plain (yellow color), North West (blue color), South West (green color), Middle South (turquoise color), Yangtze River Delta (deep pink color) and Pearl River Delta (PRD) (light pink color).

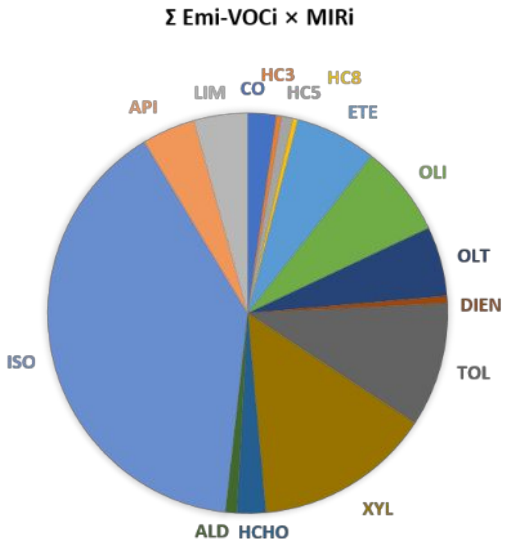

Fig. S3. Pie chart of the VOCs speciation represented by the reactivity of VOC emissions, in the form of Emi-VOCi×MIRi, as the inputs of default case in the model simulation. The VOCs components contained in each category are given in Table S1.

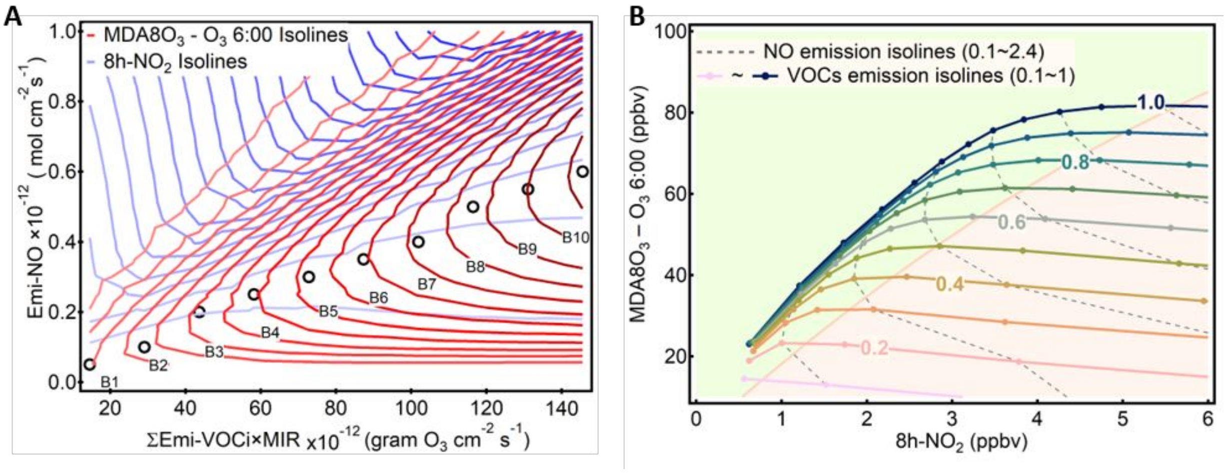

Fig. S4. (A) The modeled DPO_3 isolines and $8hNO_2$ isolines of default case. There are no multi-intersections between the DPO_3 and $8hNO_2$ isolines in the scope of the precursor settings, which means a one-to-one mapping exists between the DPO_3-$8hNO_2$ data and a definite VOCs and NOx emission couple. That is the rationale for assessing the precursor conditions based on locations of the observed data point on the diagram. (B) The zoomed relationship diagram of daytime produced O_3 (DPO_3=$MDA8O_3$-O_3 6:00) and 8h-NO_2 (same as Fig. 2A) at the lower NO_2 region.

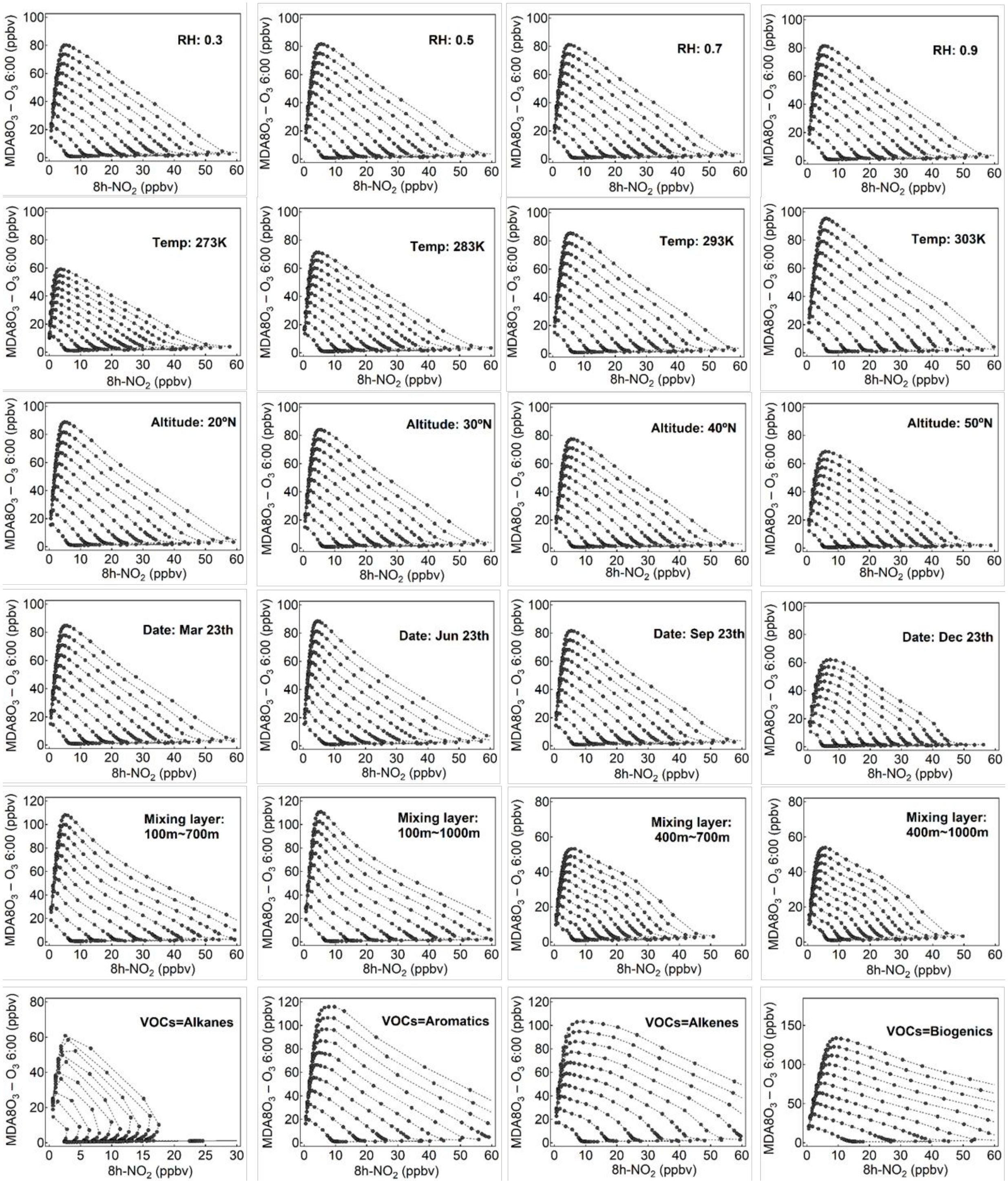

Fig. S5. The model simulated relationship diagram of DPO_3-$8hNO_2$ and VOCs emission isolines of all the examined scenarios. The different VOC scenarios were examined by setting all the VOC emissions as the target VOCs group. For example, the alkane scenario was simulated with all VOC emissions as alkanes species, and similar settings were used for the aromatics, alkanes, and biogenic scenarios.

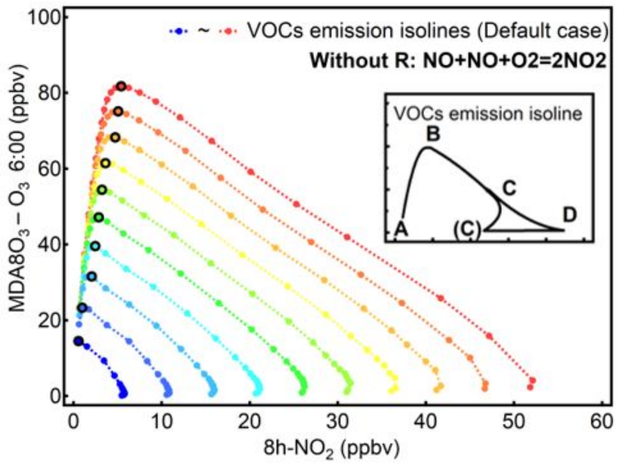

Fig. S6. VOCs emission isoline of the default case by excluding the reaction of $NO+NO+O_2=2NO_2$ in the model mechanism. Compared to Fig 2A, the VOCs isolines without this reaction bent at high NO_2 region. Except for the alkanes case, the reaction of $NO+NO+O_2=2NO_2$ is significant enough to compensate the photochemical production of NO_2 fades in the default case and all other scenarios tests.

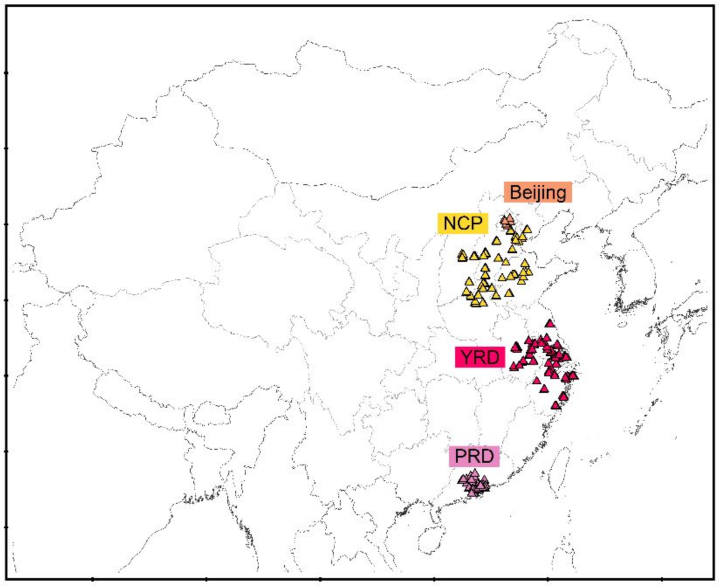

Fig. S7. The site coverage of the North China Plain (NCP), Pearl River Delta (PRD) and Yangtz River Delta (YRD) region of China in the analysis.

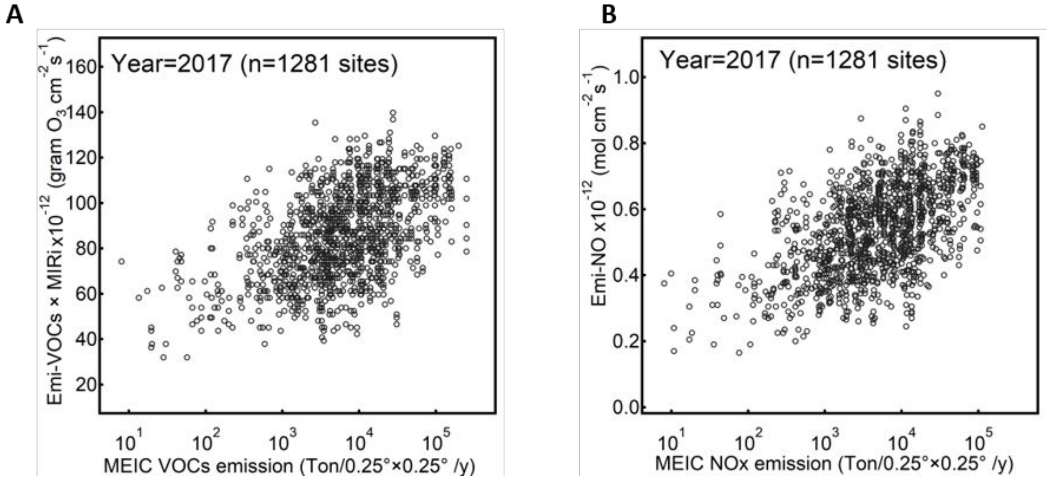

Fig. S8. The comparison of estimated (A) VOC and (B) NO_x emissions at different sites from the DPO_3-8hNO_2 diagram by referring to their nearby emission isolines with the MEIC-reported VOC and NO_x emission of the grid that the corresponding site locates.

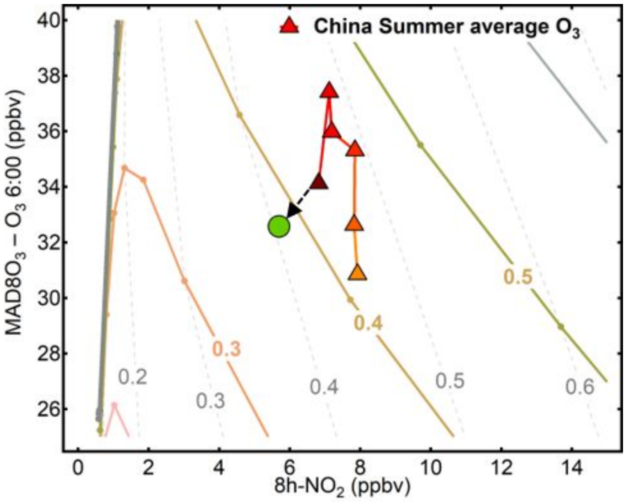

Fig. S9. The prediction of summer average change of DPO_3-$8hNO_2$ values with synergetic 10% reduction in VOC and NO emissions control in China after 2020. The summer seasonal average data is superimposed on VOC emission isolines (solid color lines) and NO emissions isolines (grey dash lines) of the modeled summer case.

Table S1.

Volume fraction settings of top 30 VOC species according to the MEIC, biogenic species and CO in the box model

MEIC top 30 VOCs species	Molecule Mass	MIR gram/gram	MEIC OFP-O3 (Gg/y)	EMIC-emission (Gg/y)	Volume fraction	RACM CATEGORY
Acetylene	26.04	0.95	696.60	730.00	1.07%	HC3
Isopentane	72.15	1.45	961.40	665.00	0.35%	HC5
N-hexane	86.18	1.24	584.90	470.30	0.21%	HC5
Butyl cellosolve	118.17	2.90	640.70	221.20	0.07%	HC8
Ethylene	28.06	9.00	10949.40	1217.30	1.65%	ETE
2-methyl-2-butene	70.13	14.08	3025.30	214.90	0.12%	OLI
Trans-2-butene	56.11	15.16	2659.60	175.40	0.12%	OLI
Cis-2-butene	56.11	14.24	2278.30	160.00	0.11%	OLI
Trans-2-pentene	70.13	10.56	1671.60	158.20	0.09%	OLI
Cis-2-pentene	70.13	10.38	1333.10	128.40	0.07%	OLI
1-Methylcyclopentene	82.14	12.49	652.20	52.20	0.02%	OLI
3-methyl-trans-2-pentene	84.16	13.17	636.90	48.40	0.02%	OLI
Propylene	42.08	11.66	4738.10	406.10	0.37%	OLT
1-butene	56.11	9.73	2601.40	267.50	0.18%	OLT
2-methyl-1-butene	70.13	6.40	768.00	120.00	0.07%	OLT
1-pentene	70.14	7.21	631.10	87.50	0.05%	OLT
Styrene	104.15	1.73	607.10	350.20	0.13%	OLT
1,3-butadiene	54.09	12.61	964.50	76.50	0.05%	DIEN
Toluene	92.14	4.00	13641.00	3406.20	1.41%	TOL
Ethylbenzene	106.17	3.04	2289.90	753.80	0.27%	TOL
Benzene	78.11	0.72	770.50	1069.20	0.52%	TOL
M & p-xylene	106.17	7.80	11245.70	1442.70	0.52%	XYL
O-xylene	106.17	7.64	5407.20	707.80	0.25%	XYL
Isomers of xylene	106.17	7.76	2179.50	280.70	0.10%	XYL
1,2,4-trimethylbenzene	120.19	8.87	2142.90	241.60	0.08%	XYL
1-Methyl-3-ethylbenzene	120.19	7.39	1045.20	141.40	0.04%	XYL
1,2,3-trimethylbenzene	120.19	11.97	923.50	77.20	0.02%	XYL
1,3,5-trimethylbenzene	120.19	11.76	838.00	71.20	0.02%	XYL
Formaldehyde	30.03	9.46	3862.80	408.60	0.52%	HCHO
Acetaldehyde	44.05	6.54	1528.60	233.80	0.20%	ALD
CO	28.00	0.06	3837.36	63955.94	86.95%	CO
ISO	68.12	10.61	65787.17	6200.49	3.47%	ISO
API	136.23	4.51	7157.19	1586.96	0.44%	API
LIM	136.23	4.55	7220.66	1586.96	0.44%	LIM

Table S2.

Modeling configuration and settings for the default case and different scenarios.

Model Setting	Default case (Different Scenarios)	
Temperature	290k (273k, 283k, 293k, 303k)	
Latitude	34°N (20°N, 30°N, 40°N, 50°N)	
Date	Sep. 23th (Mar. 23th, Jun. 23th, Sep. 23th, Dec. 23th)	
Relative humidity	RH=0.5 (RH=0.3, RH=0.5, RH=0.7, RH=0.9)	
Mixing layer height	200m~1000m (100m-700m, 400m-700m, 100m-1000m, 400m-1000m)	
Running days	5 Days	
Methane	Conc. = 1700 ppbv	
VOCs composition (volume fraction)	AVOCs referring to literature [2, 4]: HC3 (12.27%), HC5(6.42%), HC8(0.82%), ETE(18.99%), OLI(6.28%), OLT(9.08%), DIEN(0.62%), TOL(25.28%), XYL(11.96%), HCHO(5.96%), ALD(2.32%)	
	Emi BVOCs=0.5 × Emi-AVOCs, API (10.61%), LIM(10.61%), ISO(79.69%) referring to literature [7]	
	Emi-CO = 10 × Emi-AVOC	
VOCs Emission isolines (unit of Emission rates = mol·cm^{-2}·s^{-1}) (unit of ΣEmi-VOCi×MIRi = gram O_3 cm^{-2}·s^{-1})	Lines	Line1, Line2, Line3, Line4, Line5, Line6, Line7, Line8, Line9, Line10
	Emi-AVOC	2×10^{-14}~ 2×10^{-13} (unit = mol·cm^{-2}·s^{-1})
	Emi-BVOC	1×10^{-14} ~ 1×10^{-13} (unit = mol·cm^{-2}·s^{-1})
	Emi-CO	2×10^{-13} ~ 2×10^{-12} (unit = mol·cm^{-2}·s^{-1})
	ΣEmi-VOCi×MIRi	1.46×10^{-11} ~ 1.46×10^{-10} (unit = gram O_3 cm^{-2}·s^{-1})
NO Emission Isolines (unit of Emission rates = mol·cm^{-2}·s^{-1})	Sets of 24 NO emission rates, which were 5×10^{-14}, 1×10^{-13}, 1.5×10^{-13}, 2×10^{-13}, 2.5×10^{-13}, 3×10^{-13}, 3.5×10^{-13}, 4×10^{-13}, 4.5×10^{-13}, 5×10^{-13}, 5.5×10^{-13}, 6×10^{-13}, 6.5×10^{-13}, 7×10^{-13}, 7.5×10^{-13}, 8×10^{-13}, 8.5×10^{-13}, 9×10^{-13}, 9.5×10^{-13}, 1×10^{-12}, 1.1×10^{-12}, 1.2×10^{-12}, 1.8×10^{-12}, 2.4×10^{-12} (unit = mol·cm^{-2}·s^{-1})	

Table S3.

The model simulated data of daytime produced O_3 (DPO_3=MDA8O_3-O_3 6:00) and 8h-NO_2 (ppgv) under 240 settings of VOC and NOx emissions in the default case, with condition of latitude (34°N), day of the year (Sep.23th), temperature (290K), relative humidity (50%), mixing layer height (MLH, 200m-1000m). These data is the raw data used to produce the isolines plot of Fig. 2A.

			VOC emissions																			
		Isolines	VOCs-0.1		VOCs-0.2		VOCs-0.3		VOCs-0.4		VOCs-0.5		VOCs-0.6		VOCs-0.7		VOCs-0.8		VOCs-0.9		VOCs-1.0	
		Emission rate (mol/cm^2/s)	1.46E-11		2.91E-11		4.37E-11		5.82E-11		7.28E-11		8.74E-11		1.02E-10		1.16E-10		1.31E-10		1.46E-10	
NO emissions																						
Isolines	Emission rate (mol/cm^2/s)		NO_2	DPO_3	NO_2	DPO_3	NO_2	DPO_3	NO_2	DPO_3	NO_2	DPO_3	NO_2	DPO_3	NO_2	DPO_3	NO_2	DPO_3	NO_2	DPO_3	NO_2	DPO_3
NO_x-0.05	5E-14		0.56	14.49	0.62	18.90	0.67	21.32	0.70	22.67	0.70	23.33	0.69	23.57	0.67	23.57	0.65	23.44	0.63	23.26	0.62	23.05
NO_x-0.10	1E-13		1.52	13.01	1.00	23.30	1.05	28.30	1.14	31.56	1.20	33.81	1.23	35.34	1.25	36.35	1.25	36.95	1.23	37.26	1.21	37.37
NO_x-0.15	1.5E-13		3.47	9.34	1.74	22.90	1.42	31.42	1.46	36.51	1.55	40.12	1.63	42.79	1.68	44.78	1.73	46.25	1.74	47.28	1.73	47.98
NO_x-0.20	2E-13		4.64	5.37	3.78	18.71	2.08	31.56	1.85	39.11	1.88	44.17	1.96	47.92	2.03	50.83	2.11	53.10	2.15	54.86	2.18	56.19
NO_x-0.25	2.5E-13		5.29	3.13	6.31	14.44	3.62	28.44	2.47	39.57	2.27	46.49	2.28	51.45	2.35	55.26	2.43	58.30	2.49	60.74	2.56	62.71
NO_x-0.30	3E-13		5.59	2.20	8.37	9.19	6.31	24.17	3.64	37.62	2.85	47.16	2.68	53.61	2.68	58.45	2.74	62.28	2.81	65.39	2.88	67.93
NO_x-0.35	3.5E-13		5.79	1.72	9.63	5.71	9.42	18.52	5.95	33.62	3.83	46.00	3.24	54.43	3.09	60.52	3.07	65.23	3.13	69.03	3.19	72.18
NO_x-0.40	4E-13		5.95	1.43	10.30	4.06	12.22	12.41	9.09	28.81	5.66	42.87	4.09	53.80	3.62	61.45	3.48	67.23	3.46	71.82	3.48	75.59
NO_x-0.45	4.5E-13		6.16	1.24	10.60	3.16	14.02	8.13	12.88	21.84	8.54	38.46	5.56	51.58	4.41	61.19	4.01	68.26	3.88	73.78	3.84	78.25
NO_x-0.50	5E-13		6.34	1.11	10.94	2.61	14.95	5.87	16.26	15.18	12.20	32.32	8.02	47.71	5.63	59.65	4.73	68.27	4.39	74.88	4.26	80.16
NO_x-0.55	5.5E-13		6.53	1.01	11.13	2.24	15.43	4.60	18.35	10.37	16.46	24.62	11.37	42.49	7.64	56.58	5.78	67.22	5.07	75.10	4.75	81.34
NO_x-0.60	6E-13		6.80	0.94	11.31	1.97	15.80	3.80	19.59	7.62	20.23	17.60	15.56	35.05	10.64	52.01	7.45	64.92	6.02	74.43	5.42	81.74
NO_x-0.65	6.5E-13		7.10	0.89	11.58	1.78	16.18	3.25	20.28	6.01	22.74	12.42	20.29	26.97	14.49	45.40	10.04	61.04	7.42	72.74	6.27	81.34
NO_x-0.70	7E-13		7.37	0.85	11.77	1.62	16.39	2.85	20.78	4.97	24.26	9.28	24.43	19.75	19.15	37.28	13.53	55.35	9.61	69.61	7.51	80.10
NO_x-0.75	7.5E-13		7.73	0.83	12.08	1.51	16.70	2.55	21.11	4.25	25.14	7.38	27.19	14.31	24.28	28.99	17.86	47.56	12.72	64.71	9.35	77.68
NO_x-0.80	8E-13		8.08	0.81	12.40	1.42	16.88	2.32	21.53	3.73	25.79	6.13	28.74	10.86	28.61	21.62	22.93	39.16	16.68	57.62	12.06	73.56
NO_x-0.85	8.5E-13		8.50	0.79	12.66	1.34	17.20	2.14	21.73	3.33	26.21	5.25	30.02	8.70	31.50	16.04	28.41	30.73	21.45	49.29	15.66	67.33
NO_x-0.90	9E-13		8.96	0.78	13.04	1.28	17.54	1.99	22.08	3.02	26.51	4.60	30.58	7.26	33.45	12.36	33.02	23.30	26.91	40.74	20.08	59.25
NO_x-0.95	9.5E-13		9.46	0.78	13.44	1.23	17.76	1.87	22.43	2.77	26.94	4.11	31.10	6.23	34.67	9.98	36.06	17.62	32.55	32.23	25.23	50.72
NO_x-1.0	1E-12		9.94	0.78	13.79	1.19	18.14	1.77	22.61	2.57	27.14	3.72	31.70	5.46	35.60	8.35	37.93	13.76	37.36	24.80	30.99	42.08
NO_x-1.1	1.1E-12		11.11	0.79	14.75	1.14	18.83	1.62	23.36	2.26	27.90	3.15	32.42	4.41	36.64	6.31	40.36	9.41	42.69	15.08	41.75	26.15
NO_x-1.2	1.2E-12		12.43	0.81	15.75	1.10	19.77	1.51	24.02	2.05	28.47	2.76	33.01	3.73	37.46	5.10	41.85	7.14	45.39	10.44	47.20	16.32
NO_x-1.8	1.8E-12		23.01	1.02	24.73	1.14	27.43	1.32	30.68	1.56	34.30	1.85	38.22	2.21	42.37	2.64	46.70	3.17	50.80	3.82	55.27	4.64
NO_x-2.4	2.4E-12		37.12	1.28	37.97	1.33	39.40	1.42	41.59	1.54	44.27	1.70	47.09	1.89	50.43	2.12	54.04	2.38	57.54	2.68	61.54	3.04

SI References

1. Stockwell, W. R.; Kirchner, F.; Kuhn, M.; Seefeld, S., A new mechanism for regional atmospheric chemistry modeling. *Journal of Geophysical Research-Atmospheres* **1997,** *102* (D22), 25847-25879.
2. Middleton, P.; Stockwell, W. R.; Carter, W. P. L., Aggregation and analysis of volatile organic compound emissions for regional modeling. *Atmospheric Environment. Part A. General Topics* **1990,** *24* (5), 1107-1133.
3. Ganzeveld, L.; Lelieveld, J.; Roelofs, G. J., A dry deposition parameterization for sulfur oxides in a chemistry and general circulation model. *J Geophys Res-Atmos* **1998,** *103* (D5), 5679-5694.
4. Li, M.; Zhang, Q.; Zheng, B.; Tong, D.; Lei, Y.; Liu, F.; Hong, C.; Kang, S.; Yan, L.; Zhang, Y.; Bo, Y.; Su, H.; Cheng, Y.; He, K., Persistent growth of anthropogenic non-methane volatile organic compound (NMVOC) emissions in China during 1990–2017: drivers, speciation and ozone formation potential. *Atmos. Chem. Phys.* **2019,** *19* (13), 8897-8913.
5. Tong, D.; Cheng, J.; Liu, Y.; Yu, S.; Yan, L.; Hong, C.; Qin, Y.; Zhao, H.; Zheng, Y.; Geng, G.; Li, M.; Liu, F.; Zhang, Y.; Zheng, B.; Clarke, L.; Zhang, Q., Dynamic projection of anthropogenic emissions in China: methodology and 2015–2050 emission pathways under a range of socio-economic, climate policy, and pollution control scenarios. *Atmos. Chem. Phys.* **2020,** *20* (9), 5729-5757.
6. Wang, H.; Wu, Q.; Guenther, A. B.; Yang, X.; Wang, L.; Xiao, T.; Li, J.; Feng, J.; Xu, Q.; Cheng, H., A long-term estimation of biogenic volatile organic compound (BVOC) emission in China from 2001–2016: the roles of land cover change and climate variability. *Atmos. Chem. Phys.* **2021,** *21* (6), 4825-4848.
7. Guenther, A.; Hewitt, C. N.; Erickson, D.; Fall, R.; Geron, C.; Graedel, T.; Harley, P.; Klinger, L.; Lerdau, M.; Mckay, W. A.; Pierce, T.; Scholes, B.; Steinbrecher, R.; Tallamraju, R.; Taylor, J.; Zimmerman, P., A global model of natural volatile organic compound emissions. *Journal of Geophysical Research: Atmospheres* **1995,** *100* (D5), 8873-8892.
8. Chen, S.; Brune, W. H., Global sensitivity analysis of ozone production and O3–NOx–VOC limitation based on field data. *Atmospheric Environment* **2012,** *55*, 288-296.
